# Categorizing gender beyond the binary: inequalities in education from a multidimensional gender perspective

**DOI:** 10.1186/s41118-025-00271-2

**Published:** 2025-10-15

**Authors:** Sabina Bercovich Szulmajster

**Affiliations:** 1 Universitat Autònoma de Barcelona, Barcelona, Spain; 2 Center for Demographic Studies (CED-CERCA), Barcelona, Spain

**Keywords:** Education, Gender, Transgender, Gender identity, Non-binary, Gender fluid, Inequalities, LGBTQ +

## Abstract

This study seeks to gain a better understanding of gender diversity and its implications for educational inequality research by exploring how different methods of gender categorization shape analytical outcomes. Using large-scale, nationally representative data from Mexico’s National Survey on Sexual and Gender Diversity (ENDISEG), the study compares three gender categorization strategies: existing classifications of gender trajectories—cisgender, transgender, or something else—a binary/non-binary framework, and a multidimensional model that considers both gender trajectory and binary identification simultaneously. These two axes reflect key dimensions of gender normativity: alignment with sex assigned at birth and conformity to binary gender categories. Linear models are employed to assess disparities in post-secondary education completion. Results show that trans individuals face significant educational disadvantages, while non-binary identification alone appears unrelated to educational attainment. However, a more nuanced picture emerges when accounting for gender trajectory: trans non-binary individuals face the steepest penalties. These findings underline the complexity of gender categorization and the analytical value of a multidimensional approach when measuring gender diversity and understanding inequalities across gender categories.

**Supplementary Information:**

The online version contains supplementary material available at 10.1186/s41118-025-00271-2.

## Introduction

Gender inequalities have been extensively explored in the fields of population studies and sociology. While much of this research has traditionally concentrated on the distinctions between men and women, recent studies have begun to shed light on disparities between cis and trans populations (Badgett et al., 2024; Carpenter et al., 2020). However, do the categories used in these studies capture the diversity of gender experiences exhaustively? How are gender categories defined, and who do they represent? What happens to individuals who defy both cisgender norms as well as the binary understanding of gender? Is there a bigger educational penalty for those who live their lives further away from these norms?

When conducting population-focused research, it is essential to adopt methods and categories that reflect gender-expansive realities (Compton et al., 2018) to ensure that studies accurately represent diverse gender populations. Non-normative gender experiences were not taken into consideration when developing surveys until recently, when two-step questions, with one related to sex assigned at birth and another asking about gender identity, began to be present in different surveys (Baumle & Nordmarken, 2022). This allowed for studies to go a step further and look at non-cis individuals: those whose gender differed from the sex registered at birth.

Yet again, the categories available in the gender identity questions are, in general, not enough to represent all individuals’ stories (Suarez et al., 2022). For instance, non-binary identities are often unavailable as answer options. Categorizing gender is not only important to capture gender diversity empirically, but also to better understand socioeconomic inequalities experienced by groups that defy norms. This raises the question: Do results regarding socioeconomic disparities change when we employ different gender categorization approaches?

The objective of this article is to explore the influence of varying gender categorization methods on educational attainment results. Building upon insights from trans and queer studies, I aim to transcend the traditional man–woman and cis–trans dichotomies, embracing a more inclusive approach that accommodates a broader spectrum of gender experiences. I seek to address two key questions: (1) Does educational attainment differ between genders? (2) How does the way we categorize gender affect research outcomes in this regard? Educational inequalities will be analyzed using the Mexican National Survey on Sexual and Gender Diversity (ENDISEG), which includes various questions that can be used to construct gender categories in different ways (INEGI, 2021).

This study examines three different methods of categorizing gender. It begins by comparing two approaches used in previous research on educational attainment: one that looks at cis, trans, and other gender-diverse individuals, and another that compares binary and non-binary people (e.g., Carpenter et al. (2022) and Wilkinson et al. (2021)). Next, I document educational differences by considering multidimensional gender identification, combining gender trajectory—i.e., cis, trans, or something else—with binary and non-binary self-recognition, as proposed by Beischel et al.’s "3 × 3" framework (2023). Linear models are used to analyze disparities within these three methods of gender categorization in terms of educational attainment.

This study advances existing research in two ways. First, it analyzes educational attainment through the lens of queer and trans studies, exploring multiple approaches to categorizing gender identities. Owing to ENDISEG's innovative survey design, it offers a novel characterization of individuals, allowing for comparisons between trans, cis, and other gender-diverse identities, as well as binary and non-binary identifications. This approach allows for a broader comprehension of the multidimensional nature of gender, as well as allowing for an in-depth comparison of categorization methods and their relation with educational attainment. This study is the first to compare educational attainment across different ways of categorizing gender.

Second, this article departs from the predominant focus on the United States in research on non-cisgender populations venturing into the Mexican context. In Mexico, the link between socioeconomic origin and life opportunities is notably pronounced and heavily influenced by traditional gender norms (Torche, 2015). This presents an intriguing foundation for studying how access to origin resources may differ for individuals identifying in non-normative ways, potentially exacerbating disparities in life opportunities between cisgender and gender-diverse populations. Among the few studies conducted in Mexico for this population, Muñoz and colleagues (2024) found that trans individuals who were assigned male at birth have similar levels of post-secondary education as cisgender individuals, while those who were registered as females at birth and identify as trans have lower rates of post-secondary education completion. In this study, all non-cis individuals are grouped, while I will be studying differentials by gender trajectory as well as binary identification, allowing for comparison across non-cis groups. Indeed, I will argue that gender differences in education are better understood by taking a multidimensional approach to measuring gender location.

## Theoretical background

The theoretical background begins with a presentation of how gender is commonly defined following broader-than-binary perspectives, including different ways of operationalizing gender, and later moves to address studies where gender inequalities in education are analyzed from such perspectives. This section ends with a description of the Mexican context in terms of educational inequalities and gender diversity.

### What do we mean when we say gender?

Gender has been defined in various ways throughout history, with many scholars trying to put into words what this key part of our lived experience is. West and Zimmerman (1987) addressed gender as an emergent feature of social situations, with normative ideas about the attitudes and actions appropriate for each sex category. Butler (1990) understands gender as performative and fluid, in the sense of being a “copy for which there is no original”. Fausto-Sterling (2019) discusses that the concept of what counts as “normal” is dynamic across time, with individuals acquiring their gender over intersubjective experiences.

These definitions leave the door open to understanding gender as a cornerstone of our identities and the way we relate with others, while also being highly situated and dynamic. Addressing gender in our studies must then conceptualize it in a broader way than the gender/sex individuals are assigned at the moment they are born. To frame the present study, inputs from queer and trans studies (Butler, 1990; Shuster, 2020; Stryker, 2004) will be used.

Identities that challenge cisnormative understandings of gender—such as transgender, non-binary, genderqueer, gender non-conforming, and gender fluid—have become increasingly visible in recent years (Flores et al., 2016; Lagos, 2022; Richards et al., 2016). While these identities are best understood through the self-definitions of the individuals who embody them, certain terms have gained ground as ways to conceptually group diverse gender experiences.

The term trans operates both as an umbrella category for those whose gender identity does not align with their sex assigned at birth, and as a specific gender identity, often combined with other terms such as man or woman (Darwin, 2020). In this sense, trans stands in contrast to cis, which refers to individuals whose gender identity corresponds with their sex assigned at birth. However, not all individuals identify as either cis or trans. Some reject both categories or use other terms, such as genderqueer or gender fluid, to name their gender experience. To account for this diversity, Beischel et al. (2023) introduce the concept of gender trajectory, which refers to the relationship between a person’s gender identity and their sex assigned at birth. This trajectory can be: Cis, when there is alignment; Trans, when there is a divergence; Or “something else”, when neither label captures the individual’s experience.

Non-binary identification represents a distinct dimension of gender. It challenges the assumption that all gender identities must align with either “man” or “woman”, and instead highlights the diversity of experiences that exist outside or beyond this binary framework. As Richards et al. (2016) notes, *“some people have a gender which is neither male nor female and may identify as both male and female at one time, as different genders at different times, as no gender at all, or dispute the very idea of only two genders.”* This diversity, however, often comes into conflict with the binary logic embedded in institutional systems, where gender is typically registered, recognized, and regulated through male or female markers only. Even in countries with legal gender recognition, it is still common to only allow for choosing between male and female (ILGA, 2020). This institutional binarism limits access to rights and services—such as documentation, education, or healthcare—for those whose identities fall outside these normative categories (Westbrook & Schilt, 2014). Non-binary individuals are thus often rendered administratively invisible.

Despite this, most quantitative studies on gender inequalities—particularly in education—continue to treat gender as a singular axis of categorization, focusing either on the cis/trans distinction or on binary versus non-binary identities. This approach can obscure the lived experiences of individuals who, for example, identify as both trans and non-binary, or as neither cis nor trans. By adopting a multidimensional framework that considers both gender trajectory (cis, trans, or something else) and binary identification (binary or non-binary), this study aims to capture a broader range of gender experiences and examine how they relate to educational inequalities.

### Existing measures of gender identity

The question of how to capture gender in surveys is still developing and has a starting point in feminist scholars’ critiques of quantitative methods (Westbrook & Saperstein, 2015). The first step that has been conducted in recent surveys is to include two separate questions regarding sex registered at birth and gender identification, currently known as the “two-step approach”. In this survey design, gender is asked in various ways, with diverse response options: some only include men and women, while others include different ways of identification, such as trans or non-binary. Individuals whose sex assigned at birth differs from their gender identification are assumed to be non-cis and presented as trans in many studies.

Some individuals’ gender identity consists of multiple overlapping terms, and this identification can change during their life course (Ruberg & Ruelos, 2020). This illustrates gender as a multidimensional and dynamic concept, which means that multidimensional measurement methods might be better suited to study it. However, most of these surveys do not allow for selection of more than one option (Suarez et al., 2022). For instance, non-binary individuals cannot also identify as trans, even though in practice these identities frequently overlap. This constraint results in a unidimensional classification, which fails to reflect the complexity of lived gender experiences. As Darwin (2020) argues, non-binary identification can hardly be sorted neatly into man/woman and cis/trans categories: non-binary and trans identification overlap, with some non-binary individuals identifying as trans, while others do not, representing a multidimensional aspect of individuals. In this context, creating mutually exclusive categories—such as cis, trans, and non-binary—may obscure important intra-group variation and produce analytically misleading results.

Responding to this need, Beischel et al. (2023) propose a multidimensional framework that incorporates two intersecting dimensions: gender trajectory—defined as cis, trans, or neither cis nor trans—and binary identification—coded as binary, non-binary, or neither binary nor non-binary. This innovative framework, known as the "3 × 3" model, classifies gender based on these two dimensions, allowing people to identify as both non-binary and trans, for example, or even non-binary and cis. To the best of my knowledge, this novel categorization approach strategy has not yet been applied empirically in the study of educational inequalities.

In this paper, I adapt this framework to the structure of the ENDISEG survey in order to explore how intersecting dimensions of cisnormativity and binarism relate to educational outcomes. Before presenting the operationalization of this approach, the next sections provide an overview of the existing literature on gender-based educational inequalities and situate the study in the Mexican context.

### Gender-based inequalities

Individuals who depart from gender norms have always existed, but have been granted legal recognition and rights quite recently (ILGA, 2020). In spite of these developments, gender-divergent individuals are still vulnerable in terms of discrimination, harassment, and violence (Valfort, 2017; GATE, 2013; Badgett et al., 2021; Badgett et al., 2024). Based on the volume edited by Baumle and Nordmarken, “Demography of Transgender, Nonbinary and Gender Minority Populations” (2022), the available studies focusing on these populations are mainly related to health outcomes, labor market experiences and outcomes, discrimination in the workplace, and a few related to poverty. All these studies present a very unfavorable situation for gender experiences that diverge from cis-hetero norms.

Gender inequalities have been extensively explored in the field of educational attainment in population studies and sociology. While much of this research has traditionally concentrated on the distinctions between men and women, recent studies have begun to include a wider diversity of gender identities, such as trans, non-binary, and gender-nonconforming. Carpenter et al. (2020) found that transgender adults tend to achieve lower levels of education than their cisgender counterparts in the United States, with only 14% holding a college degree compared with 28% among cisgender individuals. In a later study using a nationally representative sample, Carpenter et al. (2022) found that non-cisgender individuals—those who informed a different gender than their sex assigned at birth—have lower employment rates and face higher levels of poverty and food insecurity compared to their cisgender counterparts.

In recent years, studies based on administrative data have begun to emerge (Carpenter et al., 2024; Kolk et al., 2025; Thomsen et al., 2024). These studies focus on individuals who have legally changed their gender markers and/or undergone medical transition. While valuable, they are subject to a selection bias: the populations they capture tend to reflect a more privileged subset of the transgender community—likely reflecting existing barriers to access legal and medical transition.

Despite this limitation, these studies provide a clear picture of socioeconomic inequalities, consistently showing that transgender individuals experience substantial earning penalties in contexts such as the United States (Carpenter et al., 2024). However, when it comes to educational attainment, both Carpenter (2024) and Kolk (2025) find little to no difference between transgender and cisgender individuals. As noted earlier, this likely reflects the selectivity of the samples—i.e., those who complete legal or medical transitions may already have higher levels of education, allowing them to navigate institutional systems more easily.

Wilkinson et al. (2021) utilized a broader conceptualization of gender and analyzed the educational outcomes of binary transgender, non-binary, and gender-unsure youth compared with those of cisgender youth, while also exploring the differences within the non-cisgender groups. They discovered differences in terms of educational outcomes, with binary transgender and gender-unsure youths facing disadvantages compared to their cisgender and non-binary peers.

However, none of these studies have developed a multidimensional gender perspective to address these inequalities. For example, trans non-binary experiences are hardly taken into consideration, only allowing for trans or non-binary identification separately. Adopting a multidimensional perspective is essential to understanding how different combinations of gender trajectory and binary identification interact with structural conditions—particularly within institutions like the educational system, which remain deeply shaped by cisnormative and binary logics. Individuals with non-cisgender trajectories may face barriers related to recognition, stigma, and legitimacy within educational spaces, while those who identify as non-binary may find themselves invisible or unintelligible within systems that continue to classify students, records, and facilities strictly as male or female. Exploring these dimensions jointly allows us to capture how educational inequalities are not only based on who departs from gender norms, but also on how they do so.

### Mexican context

In the Mexican context, genders that stretch beyond the male/female binary have existed since precolonial times. Some of these identities are still present in some indigenous communities, such as *muxes* in the Zapotec community (Ramirez & Munar, 2022). However, legal gender identity recognition varies across the territory, with some districts having developed special procedures to grant this right: Mexico City, Sinaloa, Nayarit, Michoacán, and Coahuila (Ortiz, 2019). In states where name and gender marker modification is possible, the main document that is rectified is the birth certificate, and the procedure is expected to be mainly administrative.

Despite the legal situation, access to name and gender rectification is still not guaranteed. In many cases, judicial processes and multiple proofs are requested for individuals who seek these procedures. In addition, access to choosing identities outside of the male and female binary is still very limited. Non-binary documentation has been provided in very few situations: the first non-binary identity document was issued judicially in Guanajuato, while Veracruz granted the first administrative recognition in 2022, followed by federal acknowledgment in 2023 with an 'X' marker on voter IDs and the country's first non-binary passport (ILGA Database, 2025).

Acceptance and recognition of non-cis identities also vary throughout society, with higher acceptance found among young people, women, and those with higher education, and higher levels of discrimination perpetuated by individuals of median age and low education (Valencia et al. 2023; Gutierrez & Rubli, 2024). These results suggest that non-cis individuals can access better environments through education, an argument that has already been made for non-heterosexual individuals (Mittleman, 2022; Boertien et al., 2024).

As Torche presented in her 2015 study on intergenerational mobility in Mexico, a strong relationship with social origin is still present in this context. There is a wide gender difference between men and women, which becomes even larger and more beneficial for married men. This suggests a patriarchal structure, where men who adhere to norms benefit from socioeconomic transfers, whereas women and single men do so at a lower level. Muñoz et al. (2024) found that trans individuals assigned female at birth were the only group with lower levels of post-secondary education completion, whereas trans individuals assigned male at birth presented similar levels to the cisgender population. Given this context, it is relevant to study how non-normative individuals relate to this situation in detail, and whether considering their identities in a multidimensional way provides a clearer picture.

## Data and methods

### Data source

The number of representative surveys and censuses inquiring about gender identity is increasing, following the legal recognition of non-cis identities in many countries. Two-step questions, with one related to sex registered at birth and another inquiring about gender identification, have been recently included in the 2020s round of censuses by countries such as Argentina, Canada, and Scotland, among others.

In Mexico, a representative survey was conducted between 2021 and 2022 to identify diversity in terms of gender and sexual identities within their population (INEGI, 2021). This survey was conducted in person using a digital questionnaire and included the use of headphones to answer sensitive topics. The use of headphones ensured privacy and sensitivity, which have been shown to be important points to consider when asking questions on gender and sexuality (Miller & Wilson, 2023). One individual per household, aged 15 or older, was randomly chosen to complete the entire individual interview. Non-responses at the household and individual levels were considered in the expansion factors to ensure representativity.

### Gender identity measures

What is novel about ENDISEG, and enables this study’s exploration of multiple gender categorizations, is its distinctive approach to collecting information on gender identity. The questionnaire first asks about sex assigned at birth, with the question: “What is your sex assigned at birth?” offering the options Male, Female, and a follow-up incisive on intersex characteristics.

Then, gender identity is assessed through two separate questions. The first one is framed as: *“Do you consider yourself…”* with five single-choice options: *Male*, *Female*, *Both of them*, *None of them*, and *Something else*. If respondents provide a gender identity that differs from their sex assigned at birth, they are presented with an additional follow-up question: *“Given that your gender is different from your sex registered at birth…”* This question includes two mutually exclusive response options:

1. *“Are you transgender or transexual”,* or
2. *“Are you queer, gender-fluid or other”.*

These two options are treated as mutually exclusive within the survey instrument, meaning respondents are required to select only one of them. While these categories are not mutually exclusive in many theoretical frameworks or community understandings—where individuals might identify as both trans and gender fluid, for example—the survey design imposes a mutually exclusive logic likely aimed at simplifying analysis.^1^ Variables related to gender identity were thus created based on the responses to these questions.

Following our main research question, three different categorization methods are studied to test whether the outcome is stable throughout these categories. It is important to note that, as is common in studies analyzing diverse gender identities, the number of individuals in some categories is small. However, the sample’s national representativeness and the survey’s design ensure that the study remains valuable despite this limitation. The significance of the models’ outcomes will be taken into special consideration given this situation.

The first categorization method relates to recent literature to study non-cis populations (Carpenter et al., 2020; Lagos, 2022). These categories are based on gender trajectory, distinguishing individuals who identify as cisgender (*n* = 40.881), transgender (*n* = 120), or as neither cis nor trans—a meaningful category that includes those identifying as gender queer, gender fluid, or in some other way^2^ (*n* = 221). To create these groups, the second question related to gender identity is used, where non-cis individuals had to choose between their identification as *transgender or transsexual* versus identifying as *queer, gender-fluid, or something else*.

The second categorization focuses on the binary dimension of gender identification to explore how identification with, or departure from, the binary system relates to educational outcomes (Wilkinson et al., 2021). This method will follow the question where individuals were asked how they consider themselves, either *male*, *female*, *both*, *none*, or *something else*. Males and females were coded as binary identifying, while those who chose both, none, or something else as their identities were coded as non-binary. For the second method of categorization, the comparison is conducted between cis binaries, non-cis binaries (*n* = 158), and non-cis non-binaries (*n* = 183).

Lastly, I develop a multidimensional classification that combines gender trajectory and binary identification, adapting the “3 × 3” framework originally proposed by Beischel et al. (2023) to the structure of the ENDISEG survey. This framework makes it possible to examine how intersecting dimensions of cisnormativity and binarism shape educational inequalities. In particular, it allows an analysis of whether non-binary individuals who also identify as trans face different disadvantages than those who identify outside both cis and trans labels. This multidimensional approach contributes to recent efforts in queer demography to move beyond current gender categorizations, and to critically engage with how survey design and categorization practices both reflect and shape the realities they aim to measure (Lagos, 2022; Westbrook & Schilt, 2014; Guyan, 2022).

The “3 × 3” framework developed by Beischel et al. (2023) combines a gender trajectory (trans, cis, or something else) and a binary (binary, non-binary, or neither binary nor non-binary) dimension. Adapted from this framework, a third method of categorization is conducted by combining both gender-related questions. Five different groups are then created: cis binary, trans binary (*n* = 58), trans non-binary (*n* = 62), diverse binary (*n* = 100), and diverse non-binary (*n* = 121). Even though this categorization does not replicate exactly the categories used in the 3 × 3 framework, the approach is comparable by incorporating the two most important features of the framework: consider both dimensions of gender trajectory and binary identification, as well as allowing for third options that are not well captured by more established categories (e.g., those who do not identify as neither cis nor trans).

Table 1 shows a cross tabulation of the frequencies with which answers to the two gender questions were given. As can be noted, many trans individuals do not identify as males or females, nor do all individuals who do not identify as cis or trans identify in a non-binary way. This justifies the need to explore different ways of grouping, as well as to critically examine current ways of categorization, as the latest literature does not fully explore the different gender identities present in these lived experiences.

**Table 1:** Number of individuals by combined gender identification

		Response to: “Do you consider yourself…”					
		Female	Male	Both	None	Other	Total
	Cisgender	22453	18428	–	–	–	40881
Response to: **“**Given that your gender is different from your sex registered at birth, are you…”	Transgender or transexual	23	35	37	8	17	120
	Diverse (Queer, gender-fluid or something else)	55	45	66	20	35	221
	Total	22531	18508	103	28	52	44189

From the collected sample, 441 individuals reported a gender identity different from their sex assigned at birth, representing approximately 1% of the total sample (*n* = 44,189). Since this study focuses on educational outcomes related to post-secondary education completion, I excluded respondents under the age of 18. The final analytical sample consequently includes 341 non-cis individuals.

Based on the information on Table 1, a detailed description of the different categorization methods can be seen. In the first method, groups are created *horizontally:* a comparison between cisgender (40881), transgender (*n* = 120), and diverse (*n* = 221) is conducted. For the second method, categories are coded from the *vertical* groups: female and male are considered binary, while the rest of the options are considered as non-binary. Cis and non-cis identification is then considered to create the 3 resulting groups: cis binary (*n* = 40881), non-cis binary (*n* = 158), and non-cis non-binary (*n* = 183). For the last categorization method, a combination of both *vertical* and *horizontal* responses is taken into consideration, resulting in 5 groups: cis binary (*n* = 40881), diverse binary (*n* = 100), trans binary (*n* = 58), diverse non-binary (*n* = 121), and trans non-binary (*n* = 62).

### Other measures and method

As non-cis individuals are highly concentrated in the age range where many people go to university (Carpenter et al., 2024), as well as having restricted access to post-secondary education in the Mexican context, having completed education above the secondary level will be used as the dependent variable. To study the differences in terms of educational attainment, a variable related to post-secondary level education completion was created and analyzed, including individuals with a technical career or program, preparatory school, bachelor's degree or undergraduate program, specialization, and a master's degree or doctorate. This information is based on the question asking *“Until which year and grade did you pass in school?”*, with ten different educational levels, going from none to master/doctorate.

Age and sex assigned at birth were added as control variables to the models in a step-by-step manner. Younger individuals generally achieve higher levels of education than older generations, and in the Mexican context, women have historically exhibited lower educational attainment. This suggests that sex assigned at birth may influence educational outcomes even among those who do not identify as women (Hausman et al., 2012). While other potentially relevant factors, such as parental education or broader socioeconomic indicators, were not available in the dataset, we consider descriptive differences between groups to be an important first step towards understanding gender differences in education.

Once the distinct ways of categorization are coded, linear probability models are used to gain a better understanding of the differentials found among the groups and across the different ways of categorization in terms of post-secondary completion (Mood, 2010).

## Results

This section presents the findings of the study, beginning with a descriptive comparative analysis between cis and non-cis populations, as well as across the multidimensional gender categorization. It then turns to the results of the OLS models, structured around the three different gender categorization methods: first, a comparison between cisgender, transgender, and individuals who identify with another gender trajectory; Second, an analysis based on binary identification, distinguishing cis binary, non-cis binary, and non-cis non-binary individuals; and finally, the outcomes derived from applying the multidimensional framework, which combines gender trajectory with binary/non-binary identification.

### Descriptive results

When descriptively comparing non-cis individuals with their cis counterparts, several key distinctions emerge (Table 2). Non-cis individuals have a lower mean age than cis individuals, and their education level is more concentrated at higher levels, whereas the education pattern is more dispersed for the cis-population. Despite similar levels of employment, non-cis individuals experience higher rates of unemployment and are more likely to have student status, potentially linked to their younger age structure. Additionally, nearly half of non-cis individuals are single, more than doubling the proportion of single cis people. However, it is crucial to note that grouping non-cis individuals into a single category may obscure important disparities among gender identities, highlighting the need for a more granular examination of gender groups.

**Table 2:** Descriptive statistics comparing cisgender and non-cisgender groups

	Cis	Non-cis
	Mean/share	Mean/share
Mean age	45.21	33.97
Gender id (Share)		
Male	0.45	0.23
Female	0.55	0.23
Both	0.00	0.30
None	0.00	0.08
Other	0.00	0.15
Sex assigned at birth (Share)		
Male	0.45	0.50
Female	0.55	0.50
Education (Share)		
None	0.05	0.02
Primary	0.23	0.13
Secondary–high school	0.50	0.62
University or more	0.22	0.23
Occupation (Share)		
Employed	0.62	0.66
Unemployed	0.02	0.04
Student	0.02	0.06
Unpaid domestic worker	0.25	0.20
Other non-employed	0.09	0.04
Partnership status (Share)		
Single	0.21	0.48
Free union	0.20	0.24
Married	0.39	0.21
Separated	0.08	0.04
Divorced	0.04	0.02
Widow	0.09	0.01
Obs. (*n*)	40.881	341

By looking at Table 3, presenting the number of individuals per combined gender location, a noteworthy image appears: binary/non-binary identification does not relate clearly to trans identification. Out of the total sample of non-cis individuals, 35% identified as trans, while 65% identified as gender queer, gender fluid, or with some other gender. Of those in this last group, 45% identify in a binary way, while 55% identify as non-binary. Trans individuals have a similar pattern, with 48% having a binary identity, and 52% a non-binary one. This picture justifies the need to address gender identity in a multidimensional way, as these identities present a complex form of lived experiences regarding their gender.

**Table 3:** Number of individuals per combined identification, non-cis population

		Coding from response to: “Do you consider yourself…”		
		Binary	Non-binary	Total
Response to: **“**Given that your gender is different from your sex registered at birth, are you…”	Trans	58	62	120
	Queer, gender fluid, or something else	100	121	221
	Total	158	183	341

To get an initial picture of the characteristics of each multidimensional group, Table 4 provides the main descriptive statistics by gender location, following a combined perspective. The table reveals several distinctions across these groups. The mean age varies significantly, with cis binary individuals being the most aged group at 45 years and diverse non-binary individuals the youngest at 32 years. Gender identity distribution shows that cis binary and gender-diverse binary groups are evenly split between male and female identification, while non-binary individuals exhibit more diverse identities, including people identifying as both man and woman, none of these, and other ways of identification. Sex assigned at birth (SAAB) proportions also differ, with a higher percentage of people assigned male at birth among gender-diverse binaries and trans non-binaries. The visibility of gender expression differs across groups: while a majority of trans non-binary individuals report expressing their gender openly, most individuals in the other gender-expansive categories do not present their gender identity publicly.

**Table 4:** Descriptive statistics comparing multidimensional gender groups

	Cis binary	Diverse binary	Diverse non-binary	Trans binary	Trans non-binary
	Mean/share	Mean/share	Mean/share	Mean/share	Mean/share
Mean age	45.21	33.85	31.90	34.02	38.16
Gender identification (Share)					
Male	0.45	0.45	–	0.60	–
Female	0.55	0.55	–	0.40	–
Both	–	–	0.55	–	0.60
None	–	–	0.17	–	0.13
Other	–	–	0.29	–	0.27
Sex assigned at birth (Share)					
Male	0.45	0.55	0.45	0.40	0.61
Female	0.55	0.45	0.55	0.60	0.39
Expression—do you openly present according to your gender? (Share)					
Yes	–	0.30	0.51	0.40	0.61
No	–	0.60	0.48	0.60	0.34
Unspecified	–	0.10	0.01	0.00	0.05
Education (Share)					
None	0.05	0.01	0.01	0.03	0.03
Primary	0.23	0.12	0.07	0.14	0.27
Secondary–high school	0.50	0.60	0.62	0.64	0.63
University or more	0.22	0.27	0.30	0.19	0.06
Occupation (Share)					
Employed	0.62	0.67	0.66	0.64	0.68
Unemployed	0.02	0.06	0.02	0.05	0.03
Student	0.02	0.05	0.11	0.03	0.00
Unpaid domestic worker	0.25	0.17	0.18	0.24	0.26
Other non-employed	0.09	0.05	0.03	0.04	0.03
Partnership status (Share)					
Single	0.21	0.34	0.62	0.34	0.55
Free union	0.20	0.32	0.19	0.26	0.19
Married	0.39	0.27	0.13	0.26	0.21
Separated	0.08	0.03	0.03	0.10	0.02
Divorced	0.04	0.01	0.02	0.03	0.00
Widow	0.09	0.03	0.00	0.00	0.03

Employment rates are similar across all groups, but those who identify as queer, gender fluid, or something else have higher student status and lower unpaid domestic worker proportions. Partnership status reveals that non-binary individuals, regardless of their gender trajectory, are more likely to be single, while cis individuals have higher marriage rates. These descriptives present the complex and varied experiences within non-cis populations, emphasizing the importance of gender categorization for understanding the differences across groups.

### First categorization: gender trajectory

**Fig. 1:**
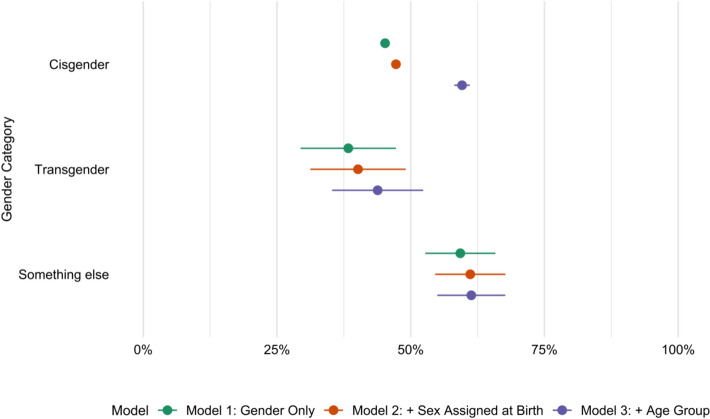
Predicted shares of post-secondary education completion, by gender trajectory. Predicted shares are taken from OLS linear probability models explaining secondary education, controlling for age and sex assigned at birth. Ref: Cis. Error bars indicate 95% confidence intervals

Using ordinary least squares linear regressions (OLS) to study educational differentials, three models are conducted: a null model considering only the effect of gender on educational attainment, a second one including sex registered at birth, and a third one considering age. Results are visualized in Fig. 1, where the predicted shares and their respective confidence intervals related to post-secondary education completion by gender group are presented, and in more detail in Table 5, where the models’ results are presented with their significance levels and standard errors.

**Table 5:** Linear regression models’ coefficients estimating the likelihood of post-secondary education attainment across gender identities (Cis, Trans, and Queer groups)

	Model 1	Model 2	Model 3
(Intercept)	0.452*** (0.00)	0.472*** (0.00)	0.162*** (0.01)
Trans	−0.069 (0.05)	−0.071 (0.05)	−0.158*** (0.04)
Queer, gender fluid, or something else	0.141*** (0.03)	0.139*** (0.03)	0.017 (0.03)
Assigned female at birth		−0.037*** (0.00)	−0.034*** (0.00)
Age group			
19–24			0.556*** (0.01)
25–29			0.479*** (0.01)
30–34			0.434*** (0.01)
35–39			0.385*** (0.01)
40–44			0.315*** (0.01)
45–49			0.272*** (0.01)
50–54			0.251*** (0.01)
55–59			0.223*** (0.01)
60–64			0.150*** (0.01)
65–69			0.109*** (0.01)

Ref. category: Cis; significance levels: ***p < 0.001; ** *p* < 0.01; *p < 0.05

In the first model, a significant positive differential is observed for those who identified as queer, gender fluid, or something else compared to the cis-population, while a non-significative penalty is observed for trans-identifying individuals. However, when considering age, the results vary significantly: the predicted probability of completing post-secondary education for cis individuals becomes higher, reaching the shares observed for those who identified as neither trans nor cis, while the penalty previously observed for trans individuals increases and becomes significant.

### Second categorization: binary dimension

For the second categorization, three groups are studied: cis binaries, non-cis binaries, and non-cis non-binaries. Men and women were coded as binary, while those who describe themselves as both man and woman, none of these, or in a different way, were coded as non-binary.

**Fig. 2:**
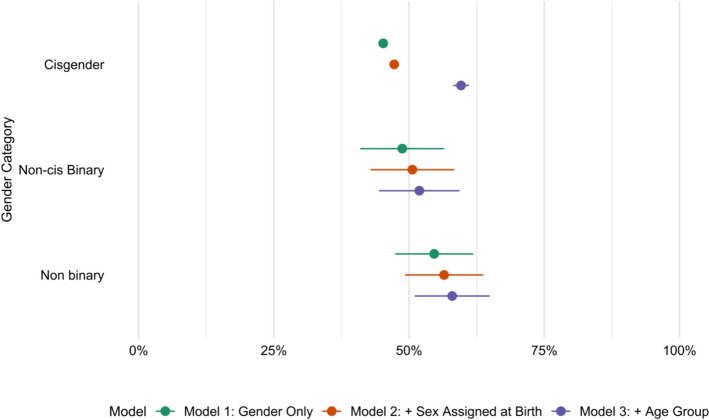
Predicted shares of post-secondary education completion, by binary dimension (Cis Binary, Non-cis Binary, and Non-cis non-binary). Predicted shares are taken from OLS linear probability models explaining secondary education, controlling for age and sex assigned at birth. Ref: Cis. Error bars indicate 95% confidence intervals

Once again, three OLS models were conducted, adding sex assigned at birth and age sequentially. As can be seen in Fig. 2, the effects of this way of categorization are almost non-significant for all the different categories. Comparing cis individuals with their non-cis binary peers, the differential becomes significant once controlling for age, with non-cis binary individuals performing worse than cis binaries. Non-binary individuals show no clear difference with the cisgender group in any of these models. Results are presented in detail in Table 6.

**Table 6:** Linear regression models’ coefficients estimating the likelihood of post-secondary education attainment across gender identities (Cis Binary, Non-Cis Binary, and Non-Cis, Non-binary groups)

	Model 1	Model 2	Model 3
(Intercept)	0.452*** (0.00)	0.472*** (0.00)	0.162*** (0.01)
Non-cis binary	0.035 (0.04)	0.034 (0.04)	−0.077* (0.04)
Non-cis nonbinary	0.094* (0.04)	0.092* (0.04)	−0.016 (0.03)
Assigned female at birth		−0.037*** (0.00)	−0.034*** (0.00)
19–24			0.557*** (0.01)
25–29			0.479*** (0.01)
30–34			0.434*** (0.01)
35–39			0.385*** (0.01)
40–44			0.314*** (0.01)
45–49			0.272*** (0.01)
50–54			0.251*** (0.01)
55–59			0.223*** (0.01)
60–64			0.150*** (0.01)
65–69			0.110*** (0.01)

Ref. category: Cis; significance levels: ***p < 0.001; **p < 0.01; *p < 0.05

### Third categorization: combined gender identification

Adopting a multidimensional understanding of gender, considering both gender trajectory and the binary/non-binary dimension, offers a clearer picture of predicted educational outcomes and potential inequalities. In the second categorization method, which considered only the binary/non-binary dimension, non-binary individuals—as a single aggregated group—showed no statistically significant effect.

**Fig. 3:**
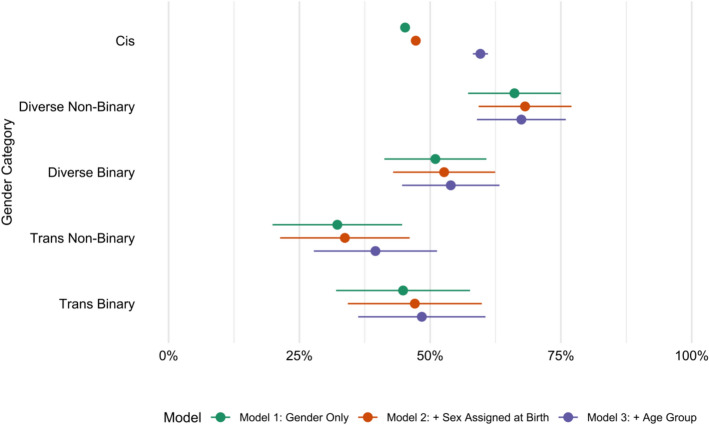
Predicted shares of post-secondary education completion, by multidimensional gender categorization. Predicted shares are taken from OLS linear probability models explaining secondary education, controlling for age and sex assigned at birth. Ref: Cis. Error bars indicate 95% confidence intervals

However, when gender trajectory is incorporated in combination with binary identification, a different pattern emerges. As shown in Fig. 3 and detailed in Table 7, only one gender group appears to be significantly penalized: trans non-binary individuals. In contrast, those who identify as non-binary but not as trans or cis (presented as *Diverse* in Fig. 3 and Table 7) show a significant positive effect, which disappears once age is accounted for—likely due to the younger age profile of this group. Among those with binary identifications, both trans binary and diverse binary individuals show negative but non-significant effects.

**Table 7:** Linear regression models’ coefficients estimating the likelihood of post-secondary education attainment across gender identities

	Model 1	Model 2	Model 3
(Intercept)	0.452*** (0.00)	0.473*** (0.00)	0.162*** (0.01)
Diverse binary	0.058 (0.05)	0.054 (0.05)	−0.057 (0.05)
Diverse non-binary	0.209*** (0.05)	0.209*** (0.05)	0.078 (0.04)
Trans binary	−0.004 (0.07)	−0.002 (0.07)	−0.112 (0.06)
Trans non-binary	−0.130* (0.06)	−0.136* (0.06)	−0.201*** (0.06)
Assigned female at birth		−0.037*** (0.00)	−0.034*** (0.00)
19–24			0.556*** (0.01)
25–29			0.479*** (0.01)
30–34			0.434*** (0.01)
35–39			0.385*** (0.01)
40–44			0.314*** (0.01)
45–49			0.272*** (0.01)
50–54			0.251*** (0.01)
55–59			0.223*** (0.01)
60–64			0.150*** (0.01)
65–69			0.109*** (0.01)

Ref. category: Cis; significance levels: ***p < 0.001; **p < 0.01; *p < 0.05

Additional analyses were conducted separately for individuals assigned female at birth (AFAB) and those assigned male at birth (AMAB). The same three categorization methods were applied, and overall, the patterns were consistent with those obtained for the full sample. However, some differences emerged. Among AFAB individuals, educational disadvantages for trans non-binary people remained significant. Among AMAB individuals, by contrast, the effect for trans non-binary people lost significance, although the coefficient remained negative. Another distinctive result is the significant penalty observed for trans binary AMAB individuals, a pattern not present in previous models, while diverse non-binary AMAB individuals showed a relative premium. Detailed results for each categorization are available in the Online Supplementary Materials.

Muñoz et al. (2024) found that AFAB individuals as a group displayed lower levels of post-secondary completion compared to cisgender individuals. Yet, inequalities differ when examined by gender identification, as the subgroup analysis reveals variation across gender groups. For AMAB individuals, Muñoz et al. reported no significant difference compared to the cisgender population. However, our disaggregated analysis shows that several inequalities do emerge, most notably the penalty affecting trans binary AMAB individuals. This highlights the value of looking at non-cis experiences by gender location rather than treating them as a homogeneous group.

## Conclusion

This study has examined educational inequalities through a multidimensional lens of gender, using a nationally representative sample from Mexico. By comparing three different categorization strategies—based on gender trajectory, binary identification, and their combination—it shows that the way gender is measured and grouped deeply influences empirical findings.

The results reveal that cis individuals consistently show higher rates of post-secondary education attainment than trans individuals, echoing previous findings (Carpenter et al., 2020), and partially in concordance with Muñoz et al. (2024), who found this disadvantage only for non-cis individuals who were registered female at birth, but not for those assigned male at birth. However, this pattern becomes more complex when non-binary identification is introduced. In the binary vs. non-binary comparison, no significant effects were found for non-binary individuals. Yet, when both gender trajectory and binary identification are considered simultaneously, important differences emerge: trans non-binary individuals face the strongest educational disadvantage, while diverse non-binary individuals (i.e., those who identify outside both cis and trans categories) show a positive effect, though this appears to be driven by a younger age structure of this group. These findings demonstrate the value of a multidimensional gender framework for uncovering intra-group variation, particularly within the broad category of non-binary.

Several limitations must be acknowledged. First, the survey design forced respondents to choose between mutually exclusive gender identity categories—transgender/transsexual vs. queer/gender-fluid/other—limiting the possibility of capturing overlapping identifications, which may have led to some ambiguity or misclassification. Second, while the ENDISEG survey offers rich and nationally representative data, the sample sizes for gender-diverse groups remain small, especially for those who identify as both trans and non-binary. This requires cautious interpretation of statistical significance and limits the generalizability of findings. Finally, the study could not account for key variables such as socioeconomic background, school trajectory, or timing of gender realization and social transition, which may condition both educational outcomes and how gender is expressed or disclosed. However, we consider descriptive differences between groups to be an important first step towards understanding gender differences in education.

These findings are partly consistent with existing literature. As in Carpenter et al. (2020) and Kolk et al. (2025), cis individuals tend to show educational advantages compared to trans individuals. However, previous studies based on administrative data in the United States found an advantage in educational attainment for transgender individuals, which may reflect selection biases, as they typically capture those who access legal or medical transition (Carpenter et al., 2024). This study, by contrast, includes a broader range of gender-diverse individuals—many of whom may not be institutionally recognized—highlighting hidden inequalities.

Wilkinson et al. (2021) found that binary trans individuals face disadvantages in educational outcomes, while non-binary individuals do not. Yet, this study shows that such a distinction may obscure important variation within the non-binary category. As Darwin (2020) and Beischel et al. (2023) argue, gender identities are not easily mapped into dichotomous frameworks, and recognizing their multidimensionality is crucial for empirical accuracy. Gender trajectories, on one hand, and binary/nonbinary relation to this trajectory on the other hand, capture distinct dimensions of gender and are related to education in different ways.

This study offers one of the first empirical applications of a multidimensional gender framework to educational inequalities in a national survey. It demonstrates that how individuals relate to both cisnormative and binary gender expectations has meaningful consequences for their access to education. Future research could explore: the timing of gender realization or transition, and how it intersects with school experiences; contextual factors, such as urban vs. rural settings, which may offer more or less space for non-normative gendered individuals; the role of socioeconomic origin, and whether it mediates or amplifies the penalties and advantages associated with gender diversity; and the inclusion of intersecting dimensions such as ethnicity, disability, and sexuality, which remain underexplored in this field. As Shuster (2020) mentions, taking into consideration multiple axes of oppression is highly needed, considering not only identification but how non-cis individuals move through life, if they can express their identities freely, and how their relation to different institutions and networks is, among other aspects of social life.

This study highlights the importance of treating gender as a multidimensional construct when analyzing social inequalities. Rather than a single identity marker, gender intersects with two axes of normativity: alignment with sex assigned at birth and conformity to binary identification. Individuals who challenge these norms—whether by identifying as trans, non-binary, or both—often face institutional barriers, particularly in educational contexts where cisnormative and binary logics prevail. The findings show that different ways of categorizing gender lead to significantly different empirical results, underscoring the need for more nuanced methodologies in population research that move beyond rigid categories and reflect the complexity of lived gender experiences.

## Supplementary Information

Supplementary material 1.

## Data Availability

The datasets analyzed during the current study are available in the INEGI repository, https://www.inegi.org.mx/programas/endiseg/2021/#microdatos.

## Footnotes

1 This limitation is important to acknowledge, as it reflects not necessarily the lived identities of respondents, but rather how survey structure constraints self-expression. It is also worth noting that these two broad categories are not widely established as mutually exclusive in Mexican LGBTQ + discourse, making the ENDISEG's wording potentially ambiguous for some participants. However, we assume here that respondents choose the answer that captures the most salient part of their identity.

2 For simplification reasons, this third gender trajectory will be presented as *diverse* when needed.
