# Supplementary material for: Categorizing gender beyond the binary: inequalities in education from a multidimensional gender perspective
Source: Genus. 2025 Oct 15;81(1):29. doi: 10.1186/s41118-025-00271-2 (PMC12528270; doi:10.1186/s41118-025-00271-2)
Supplement: Supplementary file 1 — Supplementary material 1. [file 41118_2025_271_MOESM1_ESM.docx]

## Online Supplementary Materials

In these supplementary materials, results on post-secondary education by gender identity are presented, disaggregated by sex assigned at birth. All three categorization methods were examined separately for individuals assigned female at birth (AFAB) and those assigned male at birth (AMAB). For each categorization, we present models including only gender categories as well as models additionally controlling for age (Tables A1, A2, and A3).

**First categorization: Gender trajectory**

For this categorization, the results obtained when separating the analysis by sex assigned at birth are consistent with those for the overall sample in terms of statistical significance.

**Table A1. Linear Regression Models’ Coefficients Estimating the Likelihood of Post-Secondary Education Attainment Across Gender Identities (Cis, Trans, and Queer Groups)**

|  | **AFAB** | | **AMAB** | |
| --- | --- | --- | --- | --- |
|  | **Null Model** | **+Age group** | **Null Model** | **+Age group** |
| (Intercept) | 0.435***  (0.00) | 0.123***  (0.01) | 0.472***  (0.00) | 0.169***  (0.01) |
| Trans | -0.046  (0.06) | -0.171**  (0.06) | -0.095  (0.06) | -0.146*  (0.06) |
| Queer, gender fluid, or something else | 0.132**  (0.05) | -0.002  (0.04) | 0.146**  (0.05) | 0.035  (0.05) |
| 19-24 |  | 0.570***  (0.01) |  | 0.540***  (0.02) |
| 25-29 |  | 0.475***  (0.01) |  | 0.485***  (0.02) |
| 30-34 |  | 0.445***  (0.01) |  | 0.420***  (0.02) |
| 35-39 |  | 0.397***  (0.01) |  | 0.371***  (0.02) |
| 40-44 |  | 0.327***  (0.01) |  | 0.300***  (0.02) |
| 45-49 |  | 0.280***  (0.01) |  | 0.262***  (0.02) |
| 50-54 |  | 0.265***  (0.01) |  | 0.234***  (0.02) |
| 55-59 |  | 0.208***  (0.01) |  | 0.241***  (0.02) |
| 60-64 |  | 0.151***  (0.02) |  | 0.148***  (0.02) |
| 65-69 |  | 0.106***  (0.02) |  | 0.114***  (0.02) |

*Note. Ref. Category: Cis; Significance levels: *** for p<0.001; ** for p<0.01; * for p<0.05.*

**Second categorization: Binary dimension**

Following the second categorization method, based on the binary/non-binary dimension, a negative and significant effect was found for individuals assigned male at birth (AMAB) who identify within the binary, as was observed for the sample as a whole. This effect was not observed among individuals assigned female at birth (AFAB).

**Table A2. Linear Regression Models’ Coefficients Estimating the Likelihood of Post-Secondary Education Attainment Across Gender Identities (Cis Binary, Non-Cis Binary, and Non-Cis, Non-Binary Groups)**

|  | **AFAB** | | **AMAB** | |
| --- | --- | --- | --- | --- |
|  | **Null Model** | **+Age group** | **Null Model** | **+Age group** |
| (Intercept) | 0.435***  (0.00) | 0.123***  (0.01) | 0.472***  (0.00) | 0.169***  (0.01) |
| Non-cis Binary | 0.090  (0.06) | -0.032  (0.05) | -0.024  (0.06) | -0.123*  (0.05) |
| Non-cis Non Binary | 0.053  (0.05) | -0.086  (0.05) | 0.130*  (0.05) | 0.049  (0.05) |
| 19-24 |  | 0.570***  (0.01) |  | 0.540***  (0.02) |
| 25-29 |  | 0.475***  (0.01) |  | 0.485***  (0.02) |
| 30-34 |  | 0.445***  (0.01) |  | 0.420***  (0.02) |
| 35-39 |  | 0.397***  (0.01) |  | 0.370***  (0.02) |
| 40-44 |  | 0.327***  (0.01) |  | 0.299***  (0.02) |
| 45-49 |  | 0.280***  (0.01) |  | 0.262***  (0.02) |
| 50-54 |  | 0.265***  (0.01) |  | 0.234***  (0.02) |
| 55-59 |  | 0.208***  (0.01) |  | 0.240***  (0.02) |
| 60-64 |  | 0.151***  (0.02) |  | 0.148***  (0.02) |
| 65-69 |  | 0.106***  (0.02) |  | 0.115***  (0.02) |

*Note. Ref. Category: Cis; Significance levels: *** for p<0.001; ** for p<0.01; * for p<0.05.*

**Third categorization: Combined gender identification**

The third categorization method reveals some differences compared to the full sample results. The disadvantage for trans non-binary individuals remains significant among those assigned female at birth (AFAB). However, among individuals assigned male at birth (AMAB), the effect for trans non-binary individuals is no longer significant, although the coefficient remains negative. For trans binary AMAB individuals, a significant penalty is observed, while a premium is found for diverse non-binary AMAB individuals.

**Table A3. Linear Regression Models’ Coefficients Estimating the Likelihood of Post-Secondary Education Attainment Across Gender Identities by Sex registered at birth.**

|  | **AFAB** | | **AMAB** | |
| --- | --- | --- | --- | --- |
|  | **Null Model** | **+Age group** | **Null Model** | **+Age group** |
| (Intercept) | 0.435***  (0.00) | 0.123***  (0.01) | 0.472***  (0.00) | 0.169***  (0.01) |
| Diverse Binary | 0.098  (0.07) | -0.013  (0.07) | 0.018  (0.07) | -0.092  (0.06) |
| Diverse Non-Binary | 0.156*  (0.06) | 0.005  (0.06) | 0.273***  (0.07) | 0.163*  (0.06) |
| Trans Binary | 0.079  (0.08) | -0.057  (0.08) | -0.125  (0.10) | -0.198*  (0.10) |
| Trans Non-Binary | -0.227*  (0.10) | -0.337***  (0.10) | -0.078  (0.08) | -0.115  (0.08) |
| 19-24 |  | 0.570***  (0.01) |  | 0.540***  (0.02) |
| 25-29 |  | 0.475***  (0.01) |  | 0.485***  (0.02) |
| 30-34 |  | 0.445***  (0.01) |  | 0.420***  (0.02) |
| 35-39 |  | 0.397***  (0.01) |  | 0.371***  (0.02) |
| 40-44 |  | 0.327***  (0.01) |  | 0.299***  (0.02) |
| 45-49 |  | 0.281***  (0.01) |  | 0.262***  (0.02) |
| 50-54 |  | 0.265***  (0.01) |  | 0.234***  (0.02) |
| 55-59 |  | 0.208***  (0.01) |  | 0.240***  (0.02) |
| 60-64 |  | 0.151***  (0.02) |  | 0.148***  (0.02) |
| 65-69 |  | 0.106***  (0.02) |  | 0.115***  (0.02) |

*Note. Ref. Category: Cis; Significance levels: *** for p<0.001; ** for p<0.01; * for p<0.05.*
